# Advanced Life and Its Diseases

**Published:** 1909-02-13

**Authors:** R. Douglas Powell

**Affiliations:** President of the Royal College of Physicians, Physician to the Middlesex Hospital, and Emeritus Lecturer in Medicine to the Medical School


### Hospital Clinics.

### ADVANCED LIFE AND ITS DISEASES.

By Sir R. DOUGLAS POWELL, Bart., K.C.V.O., M.D., President of the Royal College of Physicians, Physician to the Middlesex Hospital, and Emeritus Lecturer in Medicine to the Medical School.

(Being the first of three lectures delivered at the Middlesex Hospital, January 22, 1909.)

Gentlemen,—I thought it might be interesting to make a few remarks on advanced life and its diseases—respiratory, cardiac, and cerebral. Before speaking of the diseases of old age, we must come to a working definition as to what we mean by old age.

Is old age in itself a disease? I think not. Old age comes upon us not as a disease, but as a gentle and orderly atrophy of the whole organism. We are as leaves upon the tree of life, and our procession from maturity to senile decay may be compared to that attending the fall of the leaf. You will often see, if you notice much, as you walk along the woodways, that from the branches which have been diseased the leaves do not fall; they rot and wither on the stem; the ordinary changes towards death do not occur. Humphry remarks: "The march of changing events in the human body from the age of 40 or 50 to 100 is as regular, as orderly, as developmental, though less quick and therefore less apparent, as it is from birth to adolescence, or from conception to birth." When he says "it is," he means "it ought to be." In the normal descending development the relative proportions of the several organs and structures are preserved, while their forces and activity are being lowered by gradual diminution of material and nutritive activity. I must tell you, however, what you probably know already, that this is not the most ancient, nor perhaps the most modern and widespread and undisputed view of old age. The venerable Richard Mead in his book which appeared in 1795 wrote a chapter on the disease of old age, based on the views of Solomon as contained in Ecclesiastes, ch. xii. His version reads thus:

"Remember thy Creator in the days of thy youth, before the evil times come, and the years draw nigh, in which thou shalt say I find no pleasure: before the sun, and the light, and the moon, and the stars be darkened, and the clouds return after rain; when the keepers of the house shall tremble, and the soldiers shall give way, and the diminished grinders shall cease; and those that look out through holes shall be darkened; and the doors shall be shut outwardly, with a low sound of the mill, and they shall rise up at the voice of the bird: and all the daughters of music shall be of no avail; also when they shall be afraid of high places, and stumblings in the way; and the almond trees shall flower, and the Cicadæ shall come together; and the appetite shall be lost, man departing to his eternal habitation, and the mourners going about in the street: before the silver chain be broken asunder, and the golden ewer be dashed in pieces: and the pitcher be broken at the fountain head; and the chariot be dashed in pieces at the pit; and the dust return to the earth, such as it had been; and the spirit return to God who gave it."

This is a sad imagery of the waning senses, declining strength, and sinking vitality of the old. But long before and after the days of Solomon, far into, and perhaps after, the Middle Ages, the struggle for existence was more or less truly a purely physical one; and the enjoyments of life were more or less purely physical. Old age, at such a time, would naturally be regarded with a kind of at best contemptuous tolerance, only lightened in certain particular cases where the old man had passed a very strenuous youth, and had done great deeds in his time. The disabilities of age would thus be regarded as diseased conditions, and the pessimistic views of King Solomon, and the scornful kindliness with regard to old people of Shakespeare, and the monastic severity with which old people are admonished by Sir Thomas Browne are well understood in this aspect. Sir Thomas Browne makes one just and shrewd remark on old age when he says "the vital principle is consumed by length of time." Cicero takes a juster view of this period, which many of us must pass through. In his essay on old age he causes Marcus Cato to observe, "It is not likely that when the other parts of life have been well presented the last act should have been ill done, as it were by an ignorant artist." "It was necessary that there should be something final," he goes on to say; "and as in the berries of trees and the fruits of the earth, something withered and falling through seasonable ripeness." He compares the usefulness and yet apparent inactivity of the old man as like a pilot who, "holding the helm, sits in the stern at his ease." The late Sir George Humphry, who himself through the 'seventies preserved the keenest intellect and the brightest humour, and made a profound study of old age, remarks: "I often wish Shakespeare had learned to give another version of his Seven Ages, and to portray the old man, not lean and slippered, but well-preserved and booted, keen in life's interests, happy in the welfare and enjoyment of others." We all of us, I think, have met old men, and still more old women, whose age is adorned by these amiable and lovable features. One likes to see an old man booted, and perhaps spurred; but, except as a type of strenuous endurance, I do not think one would appreciate the specimen of old age recently described in the *Times*. "There was one eccentric who used to bathe in the Serpentine until he was close on 80 years of age. This old gentleman's dip was a regular performance, as well as his after-run on the shore. To see the old man go tenderly down the bank, take the gear off, place it on the edge of the lake, and plunge in, was a sight never to be forgotten. But his after-performance was the best. Out he would come, don his shoes and go for a training spin along the bank. His average time for 100 yards could possibly be beaten, but his finishing feat no other man could equal. With a leap forward, he would stand on his hands and hoist his legs—shoes and all—into the air. It might have been for the purpose of determining the flow of blood to his head, or some scientific experiment known only to himself; but he never explained!" This old gentleman was, at best, an athletic freak; he would possibly also be a very great bore. There are observances proper to age as well as to youth, and these cannot be greatly exceeded without detriment to dignity and well-being, and so should not be spoken of as examples for imitation.

When we come, finally, to the most modern view of old age, we find it again spoken of by Metchnikoff as mainly a disease. In the chapter on Old Age in his suggestive work "The Nature of Man," the argument leading to the conclusion that it is through the machinations of the bacterial flora of the large intestine that slow poisons or toxins are formed which produce arterial sclerosis in old age seems to me very inconsequent; and I would strongly advise you to accept the facts, so far as we know them, of the pathology of old age, and not to be hasty in accepting the explanations which are offered of them. It is certainly a fact that in old age parenchymatous tissues gradually dwindle, and that the less vital connective tissues increase, at least relatively, and sometimes positively. This is in accordance with the general law in pathology, that an inferior tissue will flourish with a less rich supply of blood plasma than a superior tissue. If the coronaries of the heart, for instance, are partially occluded, the muscular texture proper of the heart atrophies, and the fibrous and cellular tissue increases in place of it. It is further true that when a tissue loses its vitality it becomes vulnerable to phagocytic action, and is attacked with more or less success. But it is very much open to doubt whether, as maintained by Metchnikoff, phagocytic action is—aided by the paralysing influence of the poisons referred to—the primary event in the chain of phenomena of senile decay. Both, as far as they were operative, would seem to be rather secondary and supplementary factors to a waning vitality. Metchnikoff, as we know, regards the large intestine as the seat of this "bacterial flora," as he speaks of it, and he regards the colon as a pernicious survival in the human subject. And indeed he more than hints that the time may come when it will attract surgical attention, and that then a chief cause of senile decay may be removed from us. One longs for the genius and wit of an Arbuthnot or a Dean Swift, or even a Bernard Shaw, with adequate knowledge, to criticise this scientific study of old age presented by Metchnikoff, with all respect for his profound zoological and bacteriological knowledge. According to Fleurens the term of life of an animal is about five times the time required for full development. And by this reckoning, the term of life of a man should be 100 years. And Metchnikoff maintains that only in rare cases ought it to be of less duration than that.

Metchnikoff speaks of death, before this term at least, as unnecessary, as a disharmony, as coming before man has finished his physiological development, and when the instinct of life is still strong in him. He finds that man after maturity does not develop normally and "ends in old age and death that are premature and pathological." Nevertheless, experience will, I think, convince you that the view of Humphry is, in the main, right, that the march of events to old age and death is "one of the results of that inscrutable *vis*—call it what you will, and refer it to what you will—which determines the course and aim of each animate and inanimate object." Professor Metchnikoff describes some of the details of the process, and points out some of the means by which the changes of old age may be hastened by diseased conditions which might be prevented; but he does not attach sufficient importance to the wearing out of the mainspring of life which controls these otherwise disorderly secondary forces. If, then, you treat the manifestations of mere senility as diseased conditions you will bring discomfort to your patients and discredit to yourselves, although you may be able to quote some illustrious authors in your defence.

Old age may be said, perhaps, normally to commence at the climacteric period in men; that is to say, from about the age of 63. In women it may be ten years earlier, although their average term of life is in advance of that of men. Of 52 cases of attainment of 100 years or more recorded in a collective investigation by the British Medical Association in 1885, the males were to the females as 16 to 36. If I were asked what is the most delicate time of a man's life after puberty I should answer about the decade between 55 and 65, the decade penultimate to old age. It is then that he is most worn by the stress of life, which has not yet ended with him. It is then that he is most likely to acquire those arterial changes which shorten life. The harassment of life brings about such changes, which are greatly intensified by heredity, by gout, by early syphilis, by alcoholic excess, by excess of food, with diminishing inclination and leisure for exercise. It is then that prostatic troubles are apt to commence, and malignant disease is most often manifested. Some forms of insanity occur at this period of life, particularly dementia and general paralysis of the insane. There is a climacteric period in the male, as in the female, although less defined, and it comes about within this period, when nervous phenomena of an ill-defined sort will be often observed. In my experience, pneumonia, amongst other acute diseases such as influenza and typhoid, is prone to be of more fatal severity, and meets with a less strenuous resistance at this period of life than in older people. When a man has passed 65 all his history is written, all his creative work is done, and all his illusions are finished. He has gathered a ripe experience, an advisory capacity, a philosophic outlook that has a restful influence upon his nervous system, and gives him greater stability for a time. He has, if still sound, survived the tendency to most of the diseases which I have enumerated, and, if untouched by them, enters upon the candidature for longevity. You will therefore find that the diseases of old age—old age itself not being a disease—are for the most part those that have extended from this previous decade. Besides Old Testament examples, there are many cases recorded of great vitality extending to advanced life. I will mention, for one or two reasons, that of Luigi Cornaro, in the 14th and 15th centuries. After a somewhat exuberant youth, being of a choleric disposition and given to excesses, which brought about many illnesses, he seems to have arrived at years of discretion somewhere between the ages of 35 and 40—a little late in the day it is true!—when he had the self-command and good sense to correct and subdue his early follies. At 83 he wrote a comedy, of which he at least thought very well, and "he was able to sing with a better voice, with a louder and clearer pipe than at any period of his life." The old gentleman was evidently very pleased with himself. At 86 years of age he wrote again "On the Methods of Mending a Broken Constitution"; at 91 an "exhortation to a sober life"; and at 95 he describes himself as of much vigour and in perfect use of his faculties. He finally breathed his last, without suffering or pain, sitting in his chair, having lived over 100 years. The exhortation of Cornaro is to temperance in eating as well as in drinking. I will quote a line from him. He speaks of the Italians of the time: "Oh, wretched and unhappy Italy! Cannot you see that intemperance murders every year more of your subjects than you could lose by the most cruel plague, or by fire and sword in many battles? Those truly shameful feasts, now so much in fashion, so intolerably profuse that no tables are large enough to hold the dishes, which renders it necessary to heap them one upon another. Those feasts, I say, are so many battles; and how is it possible to live amongst such a multitude of jarring foods and disorders?"

You will find it a standing difficulty in dealing with old people that even when they themselves may be disposed to a very spare living, their friends are most solicitous to press food and drink upon them, and will endeavour to enlist your orders to aid them in doing so. Let me again quote the good sense of Cornaro, who in answer to his numerous friends, urged that as he advanced in years and lost strength, he should rather lessen than increase the quantity of food, and as the powers of the stomach grew less, the demands upon it should not be increased, and that "what we leave after a hearty meal does us much more good than what we eat." When, at 78, he was, at a weak moment, overpersuaded by his friends to partake of more than his wonted quantity of food and drink, he suffered for it by having a very severe illness, which he attributes entirely to his increased diet. I have dwelt at some length on this old man because a reference to him affords a few hints on essential points for the preservation of health in age, to which I may not have another occasion to refer. In a very interesting lecture delivered by Sir Hermann Weber before the Royal College of Physicians you will find much good advice, and an immense number of references.

We have now to speak of the heart and vessels in old age, and their changes. It is a wonderful thing, which will occur to most of you at rare intervals, to meet with a heart beating with regularity and adequate force, after unremitting labour for 75, 80, 85 and 90 years. And it is wonderful to observe the vessels themselves, even at these advanced ages, with little change; the pulse as soft and regular as would be that of a man at 45. The vessels apparently are but little damaged, and in some cases the vaso-motor mechanism is not at fault. The pulse is sometimes soft and compressible, at others hard and cord-like, according to the emotional, digestive, or other disturbance to which it is responsive. I saw yesterday an old gentleman at 95, and his pulse was as soft as that of a man in the prime of life; there was no trace to be felt in it of any degeneration. Had you been blind and judged by his pulse alone you would probably have said he was about forty, or at most fifty years of age. These conditions in old age affecting the mainspring of life—the heart and vessels—may well excite our wonder. They are exceptional in one sense, for a man, as a rule, grows old in his vessels; but they are not exceptional in another sense, for a man cannot grow very old without exceptionally good vessels; and, as these cases exemplify, vessel degeneration is no essential part of old age. I have under my occasional observation now five old people: a lady of 96, a gentleman of 95, another man of 81, another lady of 81, and another lady of 86. In none of these cases are there any signs of vascular degeneration, as far as one can recognise it by the feel of the pulse and the appearance of the patient. And in two of these cases, both of them old ladies, the vaso-motor mechanism is particularly active.

But when you meet with premature old age it is almost invariably associated with premature degenerative changes in the vascular system, which is the chief and fundamental disease of the aged. It is a very valuable clinical habit to estimate the longevity of people, a habit which Life Assurance work encourages. Apart from definite evidence of organic disease there is no index of vital endurance so valuable as the condition of the pulse, and of the heart: an estimate of the cardio-vascular system as a whole. I have often urged, in clinical teaching, that the heart is not to be listened to only with the view of hearing or not hearing murmurs, but also to ascertain the force and propriety of its mechanism; and to estimate the value of the output of force, the pulse should be felt not only for its regularity and frequency, but for its size, its compressibility, the soundness of its walls, the tension of the vessel, and approximately the general blood-pressure. You can gauge these things exactly and record them by the sphygmograph or manometer, if you are skilled in the use of these instruments; and they are valuable for teaching and recording purposes. But it is not convenient, and clearly it is not necessary if it were convenient, to carry these instruments about. Your skilled hand can recognise all these points with sufficient accuracy, and leave you much more time for the other clinical features of the case which require attention.

There is a little point which I may mention now, and that is, for a rough estimate of the blood-pressure it is better to grasp the wrist of the patient, while holding the forearm upright; then with the circumference of your fingers you can feel the pressure of the blood, and after a little practice you can estimate with considerable accuracy whether it is excessive or not.

High arterial tension is a remarkable factor in the ætiology of the discomforts, and even of the dangers, of old people, and, if I may judge from my own experience, it is a condition of much greater frequency of occurrence in old women than in old men. It is certainly rare in men of advanced life —70 or 80—although I have met with several instances of it in women. By raised arterial tension I mean a marked increase of arterial tone, a tonic spasm of the vessel. I do not think I have in mind the same cases as Sir Clifford Allbutt* alludes to in his remarks on increased blood-pressure. (Sir Clifford will not allow one to speak of arterial tension, but it is a very definite term.) He seems to regard the increased blood-pressure in such cases as generally associated with imperfect emptying of the ventricle, and dilatation and hypertrophy of the heart. The cases I am alluding to as occurring in old people are identical with similar occurrences in the young and middle-aged cases in which the arterial tension is associated with perfectly sound vessels, although in other cases the same condition of tension may be the fitful and sometimes disastrous accompaniment of various degrees of unsoundness of heart and vessels. In the view, that the condition is muscular, Dr. Savill seems, with his large experience of old people, to agree. In some cases it is attended with great discomfort and despondency, a vague uneasiness and apprehension, restlessness, and generally headache, with cold extremities. These are much the same symptoms, you will observe, as attend the same condition in the young and middle-aged. What is mere discomfort in young and middle-aged people is, however, serious, and it may be dangerous illness in old persons.

The lady I have above alluded to, who is in her ninety-sixth year, suffers occasional misery from high arterial tension, generally excited by emotion or dyspepsia. Her radial and temporal vessels, which are usually soft, and free from appreciable degeneration, become as tight as whipcord, and she suffers headache, prostration, restlessness, and general depression. Five-grain doses of phenazone given in 12 or 15 grains of effervescing citro-tartrate of soda, with a carminative, such as a teaspoonful of cognac, or some tincture of ginger, will generally, on such occasions, bring her restful sleep in a few minutes. This lady's pulse is normally soft and equable, about 78, her heart's action is regular, and its sounds well defined and unattended with murmur.

Intermittent increase of arterial tension, such as I am describing in this old lady, is generally attended with unpleasant symptoms. I have met with some cases of pure vaso-motor angina, in old people above 70, almost always women. But you will sometimes meet with an old person who has a persistently high range of arterial pressure, which is not associated with untoward symptoms. I can recall the case of an old lady considerably over 80 who is at her best and happiest with a degree of arterial pressure, which to a casual observer would be regarded as alarming.

She has a very active mind, and no doubt her cerebral vessels are good for her age, and her brain functions go on best at high pressure. She has no kidney unsoundness. A praiseworthy and orthodox attempt by the doctor to keep her tension down by enjoining a rather rigid abstinence from nitrogenous foods and the administration of alkalies, nearly brought her to her grave. Each case must, therefore, be taken on its merits: the practitioner must not only know his medicine, but he must learn his patient.

When, in an aged person, there is a liability to raised arterial pressure, you will be careful to maintain due activity of liver and bowel functions; for a loaded bowel, to which these old people are particularly liable, is one of the most usual causes of this intermittent and often dangerous discomfort. You will find great advantage from small doses of mercurial. The usual prescription I give is $\frac{1}{4}$ gr. of subchloride of mercury, and $\frac{3}{4}$ gr. blue pill. You may say, what can the odds be? They are both mercurials. I cannot explain it to you, but you will find many people cannot take larger doses of calomel, but they can take that comparatively minute dose of calomel supplemented by a small dose of another preparation of mercury, such as blue pill or the grey powder. To that you may add extract of hyoscyamus 1 gr., and you may put into the pill a grain of colocynth, or a couple of grains of dry cascara extract; or give the pill alone, and let the patient take some habitual saline aperient in the morning. These old people almost always have some favourite laxative of their own, and it is best, in prescribing for them, just to give them a dose of medicine which will affect their liver, and let them take their own aperient in addition. Of course the diet must be looked to, and if it is excessive in any way it must be properly adjusted. You will, however, be prepared frequently, as people get on beyond the 'sixties, to find some change in the condition of their vessels; the radial and temporal arteries, taken as samples, become more palpable, slightly thicker, and more defined tubes under the finger, and more visible, especially the temporal. The vessels are less elastic

* Remarks on Dr. Savill's paper on "Senile Decay." Trans. Med. Soc., vol. xx. p. 272.

and less adaptable to the lengthening and shortening of the limb; thus, when you look at the brachial artery where it is superficial on the lower arm, you note, on bending the limb, that the vessel becomes a tortuous, throbbing, sinuous tube. Corresponding to these arterial conditions, which are but examples of the whole arterial system, you find the heart's apex beat a little to the left, with the normal impulse slightly increased, the first sound a little wanting in sharpness of definition. The aortic sounds are somewhat heavy and accentuated, especially the second. With regard to the apex beat and the sounds at the apex, they are, not infrequently, obscured by some degree of emphysema in these people.

These conditions, in this degree, are not of importance, and the most that could be said of them would be that the circulation was beginning to show signs of wear. You would pass them by in silence to your patient, or you would even be justified in telling him that his heart was of an average soundness for his age. You would make a mental note, however, that he would not be amongst the candidates for *extreme* old age. This degree of senile change may be said to be more or less normal after the age of 65. Its explanation is a certain degree of atheromatous change in the vessels, which renders them less elastic, and a gradual increase in the work of the heart which causes it slightly to hypertrophy. Such a man becomes more easily leg-weary, brain-weary, heart-weary than before: he cannot do quite as much, or work at such high pressure as he could formerly, but the essential functions of mind and cerebration are as good as ever. The only advice you can give is that he should cease worrying or putting himself to fatigue, and should delegate more details of business to others. Although such persons may not become nonagenarians, they will, with care, live on to, or somewhat beyond, the allotted period. In other cases the degree of arterial thickening—indeed, of cardio-vascular change—is much more marked, and constitutes arterial sclerosis, a disease generally acquired in the period that precedes old age, and incompatible with considerable longevity. Such changes are induced or accelerated by early syphilis, alcoholism, food excesses; gout, kidney disease, and very often by a hereditary tendency. In families you often find that the people, males especially, tend to degenerate with regard to their vessels at about the same time of life. Stress of mental work and anxiety assist this in middle and later life by producing chronic high arterial tension or blood-pressure—that is, over-exertion of the cardio-vascular system—and produce hypertrophy and degenerative changes in its muscular and elastic elements. The atheroma more peculiar to old age is a superficial degenerative change in the intima of the aorta and larger vessels, and it may extend to the capillaries and minute vessels. Atheroma of the great vessels in the aged is important. The arterial wall surface is more or less interfered with and spoilt, rendered vulnerable by the atheromatous changes in it; and these surfaces are, under some circumstances, liable to bacterial invasion and ulcerative changes. I have seen such changes started in connection with oral sepsis in old people.

Atheromatous change involves an impairment of elasticity. Elasticity is, as you will remember, a very important sustaining or conservative factor of the circulation, which is automatic and distributes and maintains the systolic impulse from the heart without effort, without fatigue, and with an even current. With each degree of loss of elasticity, from cardiac change, more cardiac effort is necessary, or the blood-current becomes weaker and more jerky. When, as in normal old age, the blood-volume diminishes as these changes come on and the changes are not excessive, their mechanical results are reduced to a negligible minimum. But when the changes are disproportionate, we find symptoms of failing circulation in various arterial territories. Cramps in the leg are common in old people, especially at night; limb-weariness is experienced after slight effort, local neuralgias are frequent, and often cause considerable suffering. And when the muscular weariness affects the heart, the very citadel of life is threatened, and the end of many old people is brought about by angina pectoris or the more merciful syncopal forms of that complaint. I touched on these in my lectures last year, and I would now only remind you that these limb-wearinesses and cramps affecting the extremities of old people are mainly due to partial obstruction of the circulation, causing anæmia of muscles, which renders their nutritional changes insufficient in quantity and rapidity to maintain power.

It is very curious, but it is a clinical fact which you will often have occasion to observe, that in old people, even with considerable atheromatous change in their vessels, the muscular coats of the arterioles to a great extent escape, or, notwithstanding change, remain susceptible to vaso-motor contraction.

It is by disturbance of the cerebral area, however, that notice is generally given of a want of sustained circulation in old people: there is to be observed in many of them a tendency to giddiness on change from the recumbent to the upright posture. They experience an increasing disposition to brain fatigue, and not infrequently there is evidence of focal disturbance of circulation in the brain-centres, especially of focal anæmia in the territory of the middle cerebral artery. It is in the vessels leading to this area that atheromatous changes generally begin, and therefore under conditions of general impairment or temporary failure of circulation it is here that restriction of the current and relative anæmia occurs. I have seen many cases of transient aphasia in old people, sometimes accompanied by loss of power in the right arm, and it requires much caution at first in differentiating these cases from those of actual cerebral hæmorrhage or thrombosis. The limb symptoms to which I have alluded do not excite apprehension, and therefore attract no great attention; but these cerebral symptoms naturally cause much alarm, often more so on the part of the relatives than on the part of the patient himself. The aphasia is generally partial, and sometimes consists in using wrong words and talking nonsense, of which the patient himself is unaware, or only partially aware. After a rest in recumbency or a dose of sal volatile this will sometimes pass off, and may not recur for some time. It is commonly due to some over-fatigue or excitement.

